# The Anti-Cancer Effects of Arborinine from Ruta graveolens L. on Michigan Cancer Foundation-7 (MCF-7) Breast Cancer Cells: Inhibition of Cell Growth and Induction of Apoptosis

**DOI:** 10.7759/cureus.77985

**Published:** 2025-01-25

**Authors:** Vahid Zangouri, Hamid Zaferani Arani, Seyed Alireza Salimi Tabatabaee

**Affiliations:** 1 General Surgery, Breast Disease Research Center, Shiraz University of Medical Sciences, Shiraz, IRN; 2 General Surgery, Shiraz University of Medical Sciences, Shiraz, IRN; 3 Medicine, Kashan University of Medical Sciences, Kashan, IRN

**Keywords:** anti-tumor, apoptosis, arborinine, cytotoxic effect, mcf-7, ruta graveolens l

## Abstract

Background: Arborinine, a plant-derived alkaloid, has shown potential cytotoxic effects against various cancer cell lines. This study aims to evaluate the cytotoxicity and apoptosis effects of arborinine on breast cancer (Michigan Cancer Foundation-7 (MCF-7)) and human embryonic kidney (HEK293) as normal cell lines.

Methods: The 3-(4,5-dimethylthiazol-2-yl)-2,5-diphenyl tetrazolium bromide (MTT) assay was used to assess the inhibitory concentration of 50% (IC50) after 24 and 48 hours of treatment of HEK293 and MCF-7 cell lines with arborinine. Apoptosis was evaluated through Annexin V/PI staining, and gene expressions including BAX, BCL-2, P53, PARP, and caspases (i.e., 3, 8, and 9) were analyzed via quantitative real-time polymerase chain reaction (qRT-PCR). Additionally, intracellular reactive oxygen species (ROS) levels were measured using 2',7′-dichlorodihydrofluorescein diacetate (DCFH-DA) fluorescence.

Results: The MTT assay results indicated a dose-dependent reduction in cell viability for both HEK293 and MCF-7 cells following treatment with arborinine. The viability of HEK293 cells decreased significantly (P=0.038) at concentrations above 150 µg/mL, while IC50 for MCF-7 cells was 50 µg/mL and 25 µg/mL for 24 and 48 hours, respectively. Annexin V staining revealed apoptosis rates of 9.36% in untreated MCF-7 cells, increasing to 52.3% post-treatment. Arborinine treatment upregulated pro-apoptotic factors, including BAX, PARP, and P53, while downregulating BCL-2. Additionally, arborinine increased ROS levels by approximately 1.3-fold and decreased glutathione (GSH) levels, while enhancing superoxide dismutase (SOD) activity.

Conclusion: This study shows that arborinine reduces cell viability and induces apoptosis in MCF-7 breast cancer cells by modulating key apoptotic pathways. Its effectiveness at lower concentrations in cancerous cells highlights its potential as a promising therapeutic agent in oncology.

## Introduction

Cancer is the second leading cause of death from noncommunicable diseases, following cardiovascular issues [1]. Unfortunately, the incidence of cancer continues to rise, with an estimated 2,001,140 new cases and 611,720 deaths forecasted in the United States in 2024 [2]. While the reasons for the increase in cancer cases are complex, factors such as exposure to ionizing radiation, pollution, microorganisms, genetic predisposition, immune dysfunction, and metabolic toxins are thought to play a significant role [3-5].

Current cancer treatment strategies include surgery, chemotherapy, targeted therapy, radiotherapy, and immunotherapy [6]. However, these approaches often face significant challenges, particularly due to drug resistance, which can be intrinsic or acquired after treatment [7]. This resistance is a major contributor to cancer relapse and is the primary cause of cancer-related deaths. Consequently, the search for novel anti-cancer therapies, particularly those derived from plants, is crucial. Notably, nearly 75% of anti-cancer drugs developed between 1981 and 2014 were derived from medicinal plants [8].

Ruta graveolens L., commonly known for its use in managing pain and inflammation, has shown promise in enhancing the sensitivity of cancer cells to apoptosis while sparing normal cells [9]. For instance, it can modify the BCL-XL anti-apoptotic protein in human brain cancer cells without affecting normal B and T lymphocytes [10]. Arborinine, an important alkaloid extracted from R. graveolens L., has demonstrated the ability to inhibit the growth of crown gall tumors in vitro and has been suggested as a viable treatment for gastric and adriamycin-resistant gastric cancers [11,12]. Research indicates that arborinine suppresses ovarian cancer development and inhibits cancer cell migration while activating caspase-dependent apoptosis without causing DNA damage [13]. It appears to selectively induce apoptosis in cancer cells with minimal effects on normal cells. Furthermore, arborinine has been shown to promote apoptosis and inhibit the proliferation of immune cells, such as thymocytes and splenocytes, when stimulated with various mitogens [14,15].

Despite these promising findings, there is a notable gap in the research regarding the specific effects of arborinine on breast cancer cell lines, particularly the MCF-7 line, which is a widely used model for studying estrogen receptor-positive breast cancer. While existing studies suggest that arborinine can prevent the proliferation of various cancer cells, including HeLa and A431, its direct effects on MCF-7 cells and the underlying mechanisms of action remain poorly understood. Therefore, this study aims to investigate the effects of arborinine on cell proliferation, apoptosis, and oxidative stress in human breast cancer MCF-7 cells compared to normal cells.

## Materials and methods

Cell lines preparations

The HEK293 (a normal cell line, passage number 20) and MCF-7 (the most common breast cancer cell line, passage number 35) were purchased from the Pasteur Institute in Tehran, Iran. HEK293 cells are widely used due to their ease of transfection and their ability to grow in suspension, making them suitable for a variety of molecular biology applications. MCF-7 cells are commonly chosen for breast cancer research because they are a well-characterized model for estrogen receptor-positive breast cancer, allowing for studies on hormone responsiveness and cancer biology.

Both cell lines were maintained in high-glucose Dulbecco's Modified Eagle Medium (DMEM; PAN Biotech, Aidenbach, Germany). The medium was supplemented with 1% antibiotics (penicillin at 50 U/mL and streptomycin at 50 µg/mL) and 10% fetal bovine serum (FBS; Biowest, S1810-500, France). Approximately 106 cells were transferred into a 10 cm cell culture plate. The cells were incubated at 37ºC in a humidified atmosphere containing 5% CO_2_ for periods of 48 and 72 hours. After incubation, the cells were detached using trypsinization, and cell counts were determined using a Neubauer chamber. For cryopreservation, the cells were resuspended in DMEM containing 10% FBS and 10% dimethyl sulfoxide (DMSO). The cell suspension was aliquoted into cryovials. A controlled freezing protocol was applied, gradually reducing the temperature from -20ºC to -80ºC. Finally, the cells were transferred to liquid nitrogen at -196ºC for long-term storage.

Assessment of cell viability

To evaluate the cytotoxic effects of arborinine on the HEK293 and MCF-7 cell lines, the MTT (3-(4,5-dimethylthiazol-2-yl)-2,5-diphenyl tetrazolium bromide) assay was performed based on the study by Mirmalek et al. [16]. The MTT assay is a colorimetric method that measures cell viability based on the reduction of MTT to formazan by active mitochondria in living cells. The intensity of the formazan color is proportional to the number of viable cells.

Briefly, HEK293 and MCF-7 cells were plated in 96-well plates at a density of approximately 5,000 to 10,000 cells per well. The cells were allowed to adhere for 24 hours in a humidified incubator at 37ºC with 5% CO_2_. They were then treated with varying concentrations of arborinine for 24 and 48 hours. For controls, untreated cells were included as a baseline.

Following treatment, 10 µL of MTT solution (5 mg/mL in phosphate-buffered saline (PBS)) was added to each well. The plates were incubated at 37ºC for four hours to allow for formazan formation. After incubation, the medium was carefully removed, and 100 µL of DMSO was added to dissolve the formazan crystals. The plates were gently shaken for 10 minutes to ensure complete solubilization. The optical density (OD) was measured using an ELISA reader (Biotek ELX800 microplate reader) at a wavelength of 570 nm, with a reference wavelength of 630 nm [16].

Cell viability was calculated as a percentage relative to the untreated control group using the following formula: cell viability (%)= (OD of treated cells/OD of control cells)×100.

Each treatment was performed with three replicates to ensure statistical validity. The inhibitory concentration of 50% (IC50) dosage of arborinine on HEK293 and MCF-7 cell lines was determined as the concentration that reduced the absorbance of treated cells by 50% compared to untreated cells, using the standard curve method.

Annexin V/propidium iodide double staining and apoptosis detection

To evaluate the type of cell death induced by arborinine treatment, apoptosis was assessed using Annexin V/propidium iodide (PI) double staining, following previously published protocols [17]. MCF-7 and HEK293 cells were seeded at a density of 100,000 cells per well in 24-well plates and allowed to adhere overnight. After treatment with various concentrations of arborinine for 48 hours, both floating and adherent cells were collected and trypsinized. The harvested cells were washed twice with PBS and resuspended in 40 µL of PBS. Subsequently, 5 µL of annexin V-fluorescein isothiocyanate (FITC) (from a 50 µg/mL stock, CF488A, Biotium) and 5 µL of PI (from a 2 µg/mL stock, Biotium, 40,017) were added to the cell suspension. The samples were incubated in the dark at room temperature for 30 minutes to allow for proper staining. After incubation, the cells were washed again with PBS and resuspended in approximately 50 µL of PBS. Flow cytometric analysis was performed using a FACScan flow cytometer (BD FacsCanto II, USA) to quantify the apoptotic populations.

Evaluation of the relative expression of apoptosis-related factors

Cell Culture and Treatment Groups

MCF-7 cells were cultured in high-glucose DMEM supplemented with 10% FBS and 1% antibiotics (penicillin and streptomycin). Cells were maintained in a humidified atmosphere at 37°C with 5% CO_2_. Regarding the MTT assay, the IC50 dose of arborinine was used for the treatment of MCF-7 cells, with DMSO as the control. Upon reaching approximately 70-80% confluence, the cells were treated for 48 hours.

RNA Extraction and Complementary DNA Synthesis

After the treatment period, total RNA was extracted from the MCF-7 cells using the TRIzol reagent (Thermo Fisher Scientific) according to the manufacturer’s instructions. The quality and concentration of RNA were assessed using a NanoDrop spectrophotometer (Thermo Fisher Scientific). The Complementary DNA (cDNA) was synthesized from 1 µg of RNA using a reverse transcription kit (Thermo Fisher Scientific) following the manufacturer’s protocol.

Quantitative Real-Time Polymerase Chain Reaction

The relative expression levels of apoptosis-related factors, including BAX, BCL-2, PARP, P53, caspase 3, caspase 8, and caspase 9, were evaluated using quantitative real-time polymerase chain reaction (qRT-PCR). Specific primers for each target gene were designed, and the sequences are presented in Table 1.

**Table 1:** Primer sequences of target genes.

Genes	Primer Sequence (5' to 3')	Product Size (bp)
BAX	F:TCTCCGCCGATCCTCTC	150
	R:GCGGCCGTTGCTGATG	
BCL-2	F:GGTGGGATGACCTGGTG	200
	R:TCTCCTCCTTCTTCCGTTG	
PARP	F:GAGGACGACGACGAGGAG	120
	R:GCTGCTGCTGCTGCTGTT	
P53	F:GCCACAGGACAGGCTGAC	180
	R:GCTGCTGCTGCTGCTGTT	
Caspase 3	F:ATGACCTGGTGAGGAGGATG	130
	R:GCTGAGGAGTCTGCTGTTG	
Caspase 8	F:GCTGCTGCTGCTGCTGTT	150
	R:CCTGCTGCTGCTGCTGTT	
Caspase 9	F:GCTGCTGCTGCTGCTGTT	160
	R:CCTGCTGCTGCTGCTGTT	
GAPDH	F:GAGTCAACGGATTTGGTCGT	10
	R:GACAAGCTTCCCGTTCTCAG	

qRT-PCR was performed using SYBR Green Master Mix (Thermo Fisher Scientific) on a real-time PCR system (e.g., Applied Biosystems or StepOnePlus). The thermal cycling conditions included an initial denaturation at 95°C for 10 minutes to ensure complete denaturation of the cDNA, followed by 40 cycles of amplification. Each cycle consisted of denaturation at 95°C for 15 seconds to separate the DNA strands, and annealing and extension at 60°C for one minute, allowing the primers to bind to the target sequences and the SYBR green dye to bind to the double-stranded DNA. After amplification, a melting curve analysis was performed to verify the specificity of the amplified products by gradually increasing the temperature and monitoring the fluorescence.

The relative expression of apoptosis-related genes was analyzed using the ΔΔCT method, where ΔCT is the difference in threshold cycle (CT) number between the target genes and the housekeeping gene (e.g., GAPDH and β-actin). Data were expressed as fold changes relative to the control group, allowing for a clear comparison of gene expression levels.

Determination of Intracellular Reactive Oxygen Species Production

To evaluate the intracellular oxidative stress induced by arborinine treatment, the levels of ROS, superoxide dismutase (SOD), and glutathione (GSH) were assessed using a spectrofluorometric microplate assay [18]. MCF-7 cells were trypsinized and subsequently washed with PBS to remove any residual media. A total of 5×104 cells were resuspended in PBS to a final volume of 100 µL. The cells were then incubated with 10 µM of 2',7′-dichlorodihydrofluorescein diacetate (DCFH-DA; Sigma, D6883) for 30 minutes at 37ºC in the dark. Following incubation, the cells were washed twice with PBS to remove excess DCFH-DA. Subsequently, the cells were seeded into a 96-well plate (Thermo M33089) under dark conditions to prevent any photobleaching of the fluorescent signal. The fluorescence intensity of each well was measured using a spectrofluorometer (FLx800, BioTek Instruments Inc., USA) at excitation and emission wavelengths of 485 nm and 535 nm, respectively.

Ethical approval

The protocol was approved by the Ethics Committee of Shiraz University of Medical Sciences (IR.SUMS.MED.REC.1403.291).

Inclusion/exclusion criteria

To ensure the authenticity of the recruited cell lines, both cell lines were purchased from the Pasteur Institute of Iran, and the institution confirmed the morphology and other characteristics of the cell lines. Inclusion criteria for the study included using cells that were free from contamination and characteristic of their respective phenotypes. Exclusion criteria involved discarding any cultures that exhibited signs of contamination (e.g., turbidity and unexpected color change) or abnormal growth patterns that deviated from typical cellular morphology.

Statistical analysis

All experiments were conducted in triplicate to ensure the reliability of the results and to allow for appropriate statistical analysis. The data obtained from the cell viability assays (MTT assay), apoptosis detection (annexin V/PI staining), and intracellular ROS levels were expressed as mean ± standard deviation.

To compare the cytotoxic effects of arborinine between HEK293 and MCF-7 cell lines, statistical significance was determined using one-way analysis of variance (ANOVA). This method is suitable for comparing means among multiple groups, enabling us to assess whether there were statistically significant differences in cytotoxicity across different concentrations of arborinine. Following ANOVA, Tukey's post hoc test was employed for pairwise comparisons. This test is ideal in this context because it controls for type I errors while allowing comparisons between every pair of group means. This is particularly important when multiple comparisons are made, ensuring that we precisely identify which specific concentrations differ significantly after finding overall significant effects.

The IC50 values were calculated using nonlinear regression analysis conducted with GraphPad Prism 8 (GraphPad Software Inc., San Diego, CA). A sigmoidal dose-response model was applied for the analysis, and the fit quality was assessed using the coefficient of determination (R²), which was ≥0.95, indicating a good fit to the data.

The differences in apoptosis rates, relative expression, and oxidative stress levels between treated and untreated cells were analyzed using an independent t-test. A two-tailed p-value of less than 0.05 was considered statistically significant, allowing for the detection of differences in both directions. All statistical analyses were performed using GraphPad Prism 8.

## Results

Effects of arborinine on cell viability and apoptosis rates in HEK293 and MCF-7 cell lines

**Figure 1:**
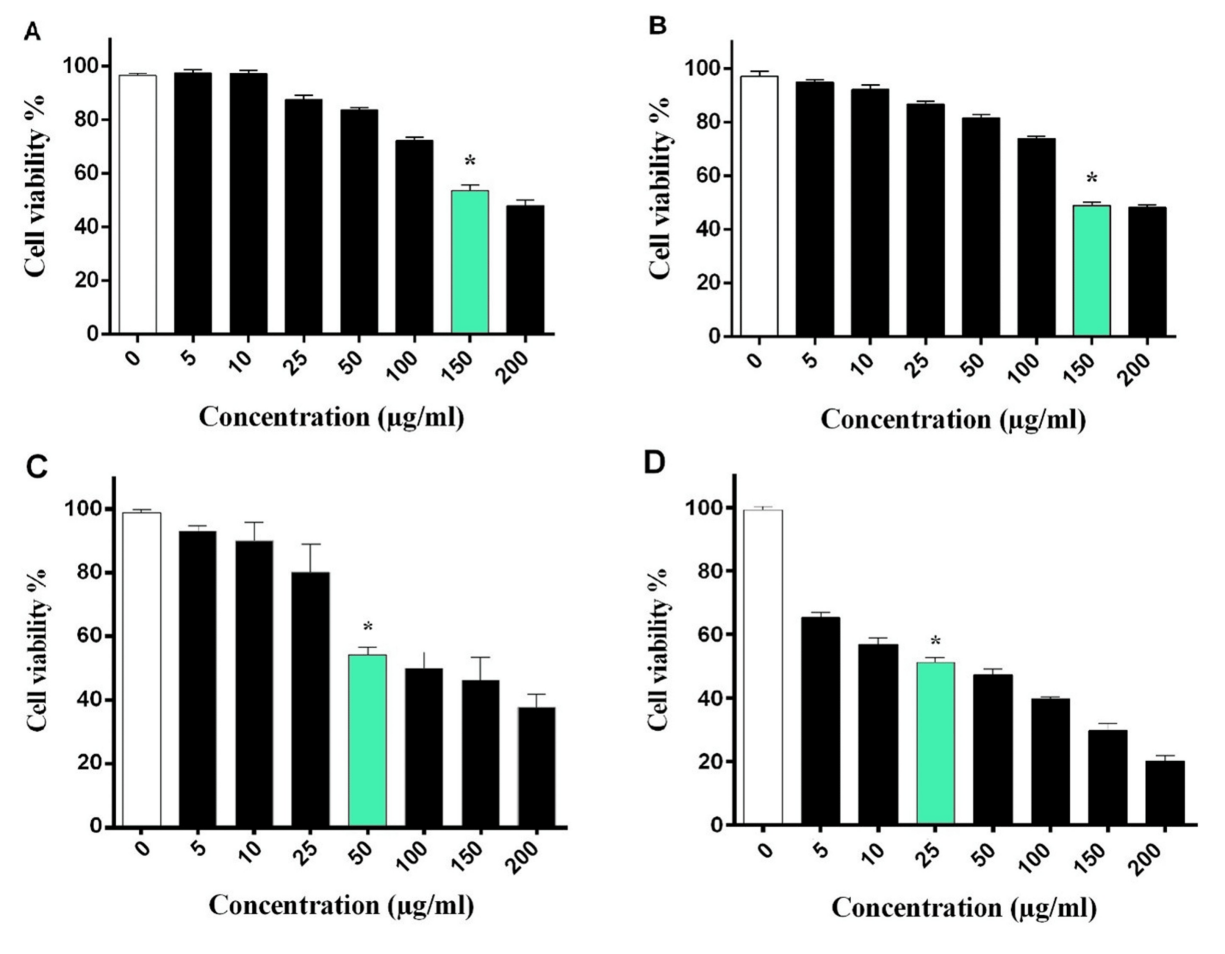
Effect of arborinine on cell viability in HEK293 and MCF-7 cells. The percentage of viable HEK293 cells after 24 (A) and 48 hours (B) of treatment with various concentrations of arborinine indicates that the IC50 of arborinine was 150 µg/mL. However, the IC50 of arborinine for MCF-7 cells after 24 (C) and 48 hours (D) were 50 and 25 µg/mL, respectively. Statistical analysis indicates significant differences (*P<0.05) compared to untreated cells.

The MTT assay was employed to evaluate the cytotoxic effects of arborinine on HEK293 and MCF-7 cell lines. Following treatment with various concentrations of arborinine, cell viability was assessed in comparison to DMSO-treated control cells. The results indicated a reduction in cell viability in both cell lines, with arborinine demonstrating a dose-dependent cytotoxic effect. Specifically, HEK293 cells exhibited decreased viability at concentrations of arborinine above 150 µg/mL after 24 and 48 hours of exposure (P=0.003; Figure 1A, 1B). In MCF-7 cells, a major decrease in viability was observed at a concentration of 50 µg/mL after 24 hours (P<0.001, Figure 1C) and at 25 µg/mL after 48 hours (P=0.021, Figure 1D). Indeed, the concentration of 50 µg/mL and 25 µg/mL of arborinine were considered as the IC50 of arborinine for 24 and 48 hours, respectively. Hence, these findings suggest that arborinine exerts significant cytotoxic effects on both HEK293 and MCF-7 cells, with a lower concentration required to induce cytotoxicity in the MCF-7 breast cancer cell line.

**Figure 2:**
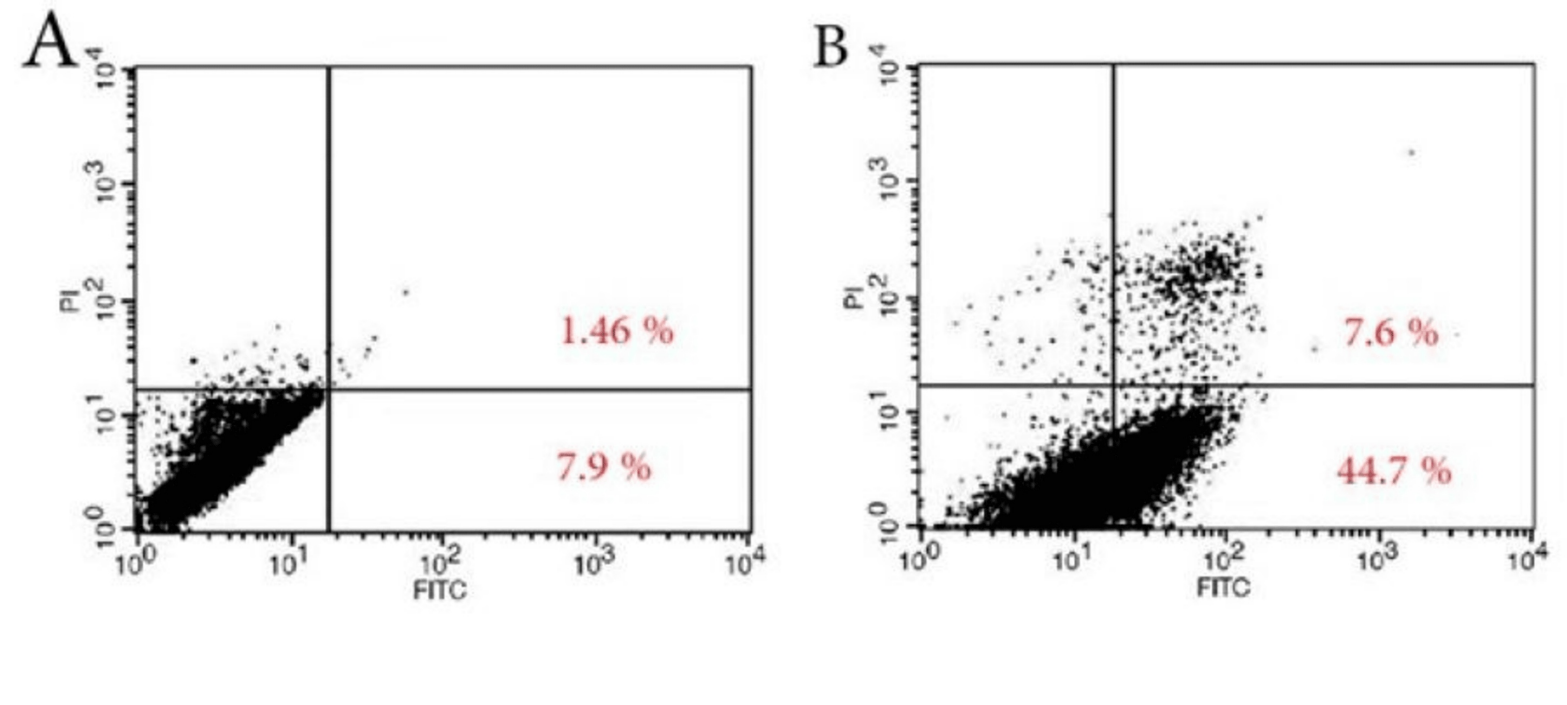
Annexin V staining in MCF-7 cells with no treatment (A) and treated with arborinine (B). PI, propidium iodide; FITC, fluorescein isothiocyanate

As illustrated in Figure 2, Annexin V staining demonstrated distinct apoptotic profiles in MCF-7 cells, both untreated and treated with arborinine. The apoptosis rates were 9.36% for untreated cells and 52.3% for treated cells.

Arborinine-induced alterations in apoptotic signaling in MCF-7 cells

**Figure 3:**
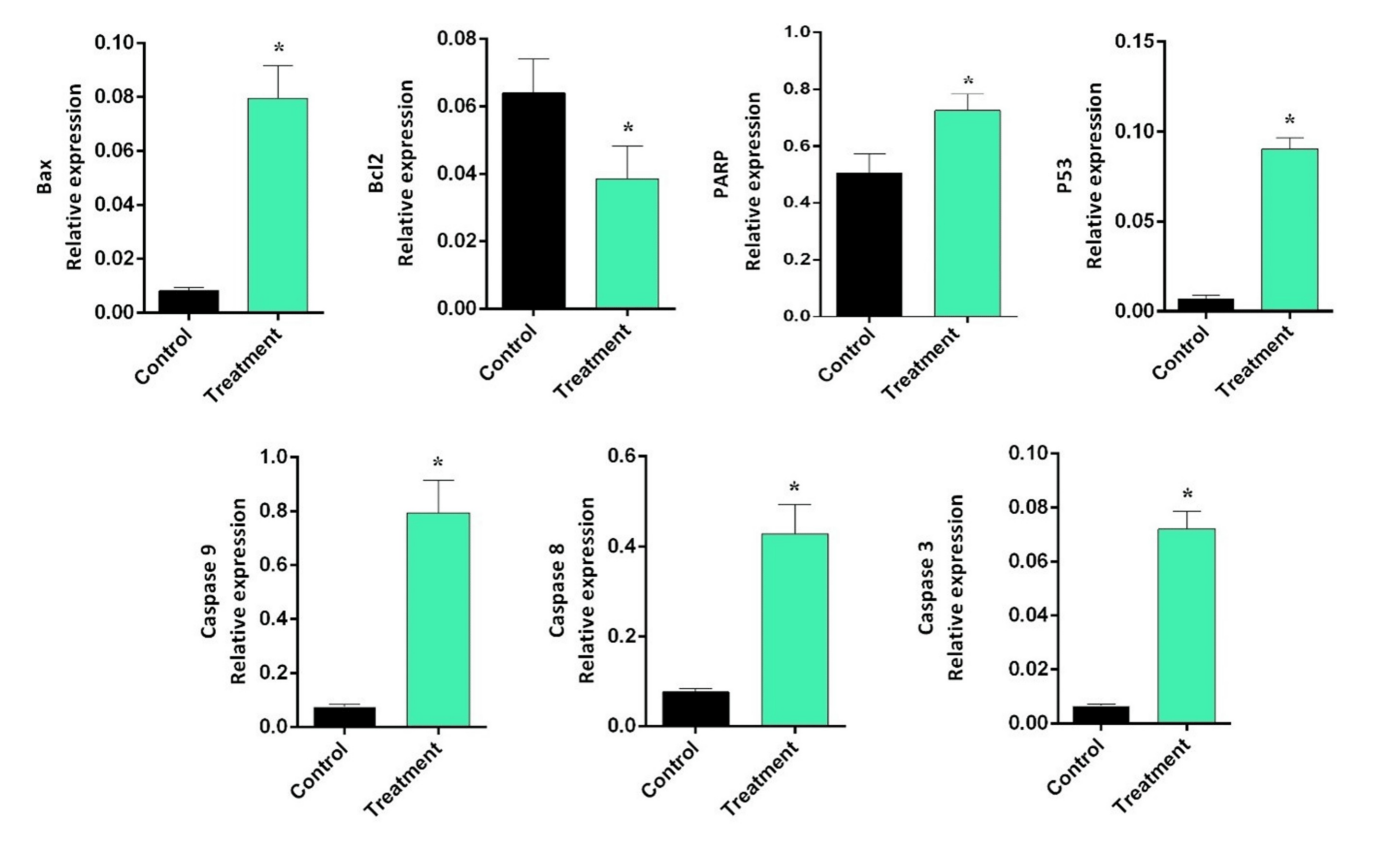
The relative expression ratio of apoptosis-related factors (BAX, BCL-2, PARP, P53, caspase 3, caspase 8, and caspase 9) in MCF-7 cells after 48 hours, induced by arborinine at a concentration of 25 µg/mL. *P<0.05 compared to control.

To evaluate the effects of arborinine on apoptosis-related factors, we quantified the relative expression levels of key proteins, including BAX, BCL-2, PARP, P53, and the caspases (3, 8, and 9), in MCF-7 cells treated with 25 µg/mL of arborinine, corresponding to the determined IC50 dose. Our analysis demonstrated a statistically significant increase in BAX expression in arborinine-treated cells compared to the untreated control group (P<0.001; Figure 3). In contrast, treatment with arborinine resulted in a statistically significant reduction in BCL-2 expression (P=0.005), indicating a shift toward pro-apoptotic signaling pathways. Furthermore, arborinine treatment significantly elevated the expression levels of PARP and P53 when compared to the non-treated control group (P<0.001). Importantly, the expression of all assessed caspases (3, 8, and 9) was also significantly increased following arborinine treatment (P=0.041). These results collectively indicate that arborinine promotes apoptosis in MCF-7 cells by upregulating pro-apoptotic factors such as BAX, PARP, and P53, while concurrently downregulating the anti-apoptotic factor, BCL-2 (Figure 3).

Arborinine-induced changes in reactive oxygen species production and antioxidant capacity in MCF-7 cells

**Figure 4:**
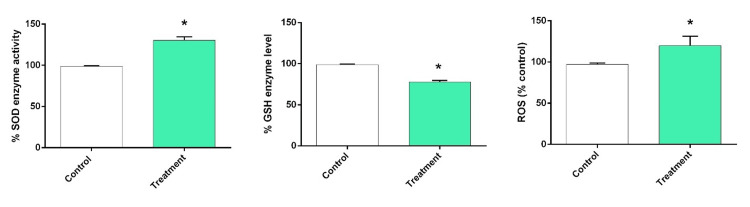
Relative expression ratios of oxidative and anti-oxidative factors in MCF-7 cells after 48 hours of treatment with arborinine at 25 µg/mL. The levels of SOD, GSH, and ROS were measured. SOD, superoxide dismutase; GSH, glutathione; ROS, reactive oxygen species *P<0.05 denotes statistical significance when compared to the control group.

To evaluate the impact of arborinine on oxidative stress, we assessed the activity of SOD, the levels of GSH, and the production of ROS in MCF-7 cells treated with 25 µg/mL of arborinine. Our findings indicated that treatment with arborinine resulted in a significant increase in ROS levels compared to the untreated control group (P=0.013) (Figure 4). Specifically, ROS levels in the arborinine-treated cells were approximately 1.3-fold higher than those in the control group, highlighting the oxidative stress-inducing effects of arborinine. In parallel, we observed a statistically significant reduction in GSH levels in the arborinine-treated cells (P=0.007). Interestingly, despite the increase in oxidative stress as indicated by elevated ROS levels, SOD activity was significantly enhanced in the arborinine-treated cells (P<0.001). This increase in SOD activity may represent a compensatory response to the heightened oxidative stress conditions induced by arborinine treatment. Hence, these results suggest that arborinine exacerbates oxidative stress in MCF-7 cells by elevating ROS levels while concurrently diminishing the antioxidant capacity through reduced GSH levels.

## Discussion

Our findings demonstrate that arborinine exerts a significant cytotoxic effect on both HEK293 and MCF-7 cell lines. Specifically, arborinine concentrations of 150 µg/mL and above were associated with reduced cell viability in HEK293 cells after both 24 and 48 hours of exposure. In MCF-7 cells, a concentration of 50 µg/mL was sufficient to decrease cell viability within 24 hours, while a concentration of 25 µg/mL impacted cell viability after 48 hours. These results suggest that arborinine may induce apoptosis and increase cell death through cytotoxic mechanisms in both cell lines.

Additionally, our study observed that arborinine treatment in MCF-7 cells led to a significant increase in the expression ratio of the pro-apoptotic factor BAX, a decrease in the anti-apoptotic factor BCL-2, and an enhancement of P53 and PARP expression compared to the untreated control group. This molecular profile indicates that arborinine mediates its cytotoxic effects by promoting apoptotic signaling pathways. The upregulation of caspases 3, 8, and 9 further supports the notion that arborinine facilitates apoptosis in MCF-7 cells through the intrinsic apoptotic pathway.

We also report that arborinine treatment resulted in increased ROS production and decreased GSH levels, highlighting its role in exacerbating oxidative stress while attenuating antioxidant capacity. The observed elevation in SOD activity may represent a compensatory response to the increased oxidative stress introduced by arborinine, suggesting that while the compound increases oxidative damage, the cells attempt to mitigate this stress.

Medicinal plants, including R. graveolens, from which arborinine is derived, have been recognized for their potential as adjuvants in the treatment of various diseases, including cancer [19-22]. Previous studies have demonstrated the anti-tumor effects of several medicinal plants on various human-derived tumor cell lines [23]. R. graveolens, in particular, is known for its broad range of therapeutic applications, including its anti-cancer properties [24-29]. Its constituent alkaloids, particularly acridones like arborinine, have shown promising anti-proliferative effects against various cancer types [30-33].

While our results affirm arborinine's potential as a therapeutic agent against cancer, they also raise concerns regarding its cytotoxicity toward normal cell lines. Studies suggest that the cytotoxic properties of R. graveolens are associated with DNA damage and chromosomal aberrations [34-36].

Hence, when comparing arborinine's efficacy with other established anti-cancer agents, such as doxorubicin and cisplatin, it is noteworthy that these agents often exhibit significant systemic toxicity and resistance issues. Doxorubicin, for example, is associated with cardiotoxicity, while cisplatin frequently leads to nephrotoxicity [37]. In contrast, the findings from our study suggest that arborinine may exert its cytotoxic effects through a mechanism that involves the modulation of oxidative stress and apoptosis without the severe side effects typically associated with conventional chemotherapeutics.

Furthermore, compounds derived from R. graveolens, including alkaloids, can generate ROS and induce cellular apoptosis, thereby contributing to their cytotoxic effects [38-39].

Indeed, the increase in ROS production, coupled with the decrease in GSH levels, indicates that arborinine enhances oxidative stress in MCF-7 cells. This mechanism is similar to that of other natural compounds, such as quercetin, which has been shown to increase ROS levels and induce apoptosis in cancer cells [40].

Although previous research indicates that arborinine exhibits greater toxicity toward tumor cells compared to normal cells [14], more extensive investigations are needed to fully ascertain its effects on normal cell lines.

In our study, higher concentrations of arborinine were necessary to elicit cytotoxic effects in normal cells compared to cancerous cells. These findings suggest that arborinine may have therapeutic utility as an anti-tumor agent at doses that might limit toxicity to normal tissues. However, the challenge of maintaining selectivity and minimizing potential harm to healthy cells is a significant consideration in drug development.

The apoptotic mechanism by which arborinine operates seems to be multifaceted; our data demonstrate an increase in apoptosis markers, including pro-apoptotic factors such as BAX and caspase activation, alongside a decrease in BCL-2 expression levels. Notably, P53 has been implicated in inducing cellular responses to genomic stress through mechanisms that promote apoptosis or cell cycle arrest [41-44]. Our findings indicating elevated P53 levels corroborate its role as a critical tumor suppressor. The activation of PARP, a key enzyme involved in DNA repair, further aligns with the notion that arborinine enhances apoptotic signaling by overwhelming the DNA repair processes, leading to increased apoptotic cell death [45-51].

Study limitations

Despite these promising findings, several limitations must be acknowledged. First, the assessment was conducted primarily in vitro, and further studies are necessary to evaluate the efficacy and safety of arborinine in vivo. Second, a detailed examination of the long-term effects of arborinine on normal cell lines has yet to be performed, with more comprehensive toxicity assessments required to determine its therapeutic index. Finally, while our study focused on specific apoptotic markers, exploring other signaling pathways and potential synergistic effects with other therapeutic agents would provide a more robust understanding of arborinine's mechanism of action.

Overall, while arborinine showcases significant anti-tumor activity, further research into its safety profile and molecular mechanisms underlying its effects in both cancerous and normal cells will be essential for its development as a therapeutic agent.

## Conclusions

The present study provides evidence that arborinine functions as a potential anti-tumor agent, effectively reducing cell viability and promoting apoptosis in the MCF-7 breast cancer cell line. Through the modulation of critical apoptosis pathways, characterized by upregulation of caspases, PARP, and P53, alongside downregulation of BCL-2, arborinine demonstrates its potential efficacy in cancer treatment. Notably, its cytotoxic effects were observed at lower concentrations in cancerous cells compared to normal cells, which could position arborinine as a viable therapeutic option in oncology.
